# Surgical Outcomes of Thyroidectomy in Geriatric Patients Aged 80 Years and Older: A Single-Center Retrospective Cohort Study

**DOI:** 10.3390/medicina60091383

**Published:** 2024-08-23

**Authors:** Wei Huang, Yi-Ju Chen, Wei-Hsin Chen

**Affiliations:** Division of General Surgery, Department of Surgery, Taichung Veterans General Hospital, Taichung 407219, Taiwan; j300521@gmail.com (W.H.);

**Keywords:** frailty, geriatric, multinodular goiter, thyroid cancer, thyroidectomy

## Abstract

*Background and Objectives*: As the global aging population grows, the incidence of thyroidectomy in elderly patients is increasing. This study aimed to evaluate the surgical outcomes of thyroidectomy in patients aged 80 years and older. *Materials and Methods*: All patients aged 80 years and older who underwent thyroidectomies at our hospital between January 2015 and December 2022 were reviewed in this retrospective cohort study. Collected data consisted of patients’ clinical characteristics, functional status, compression symptoms, preoperative assessments, perioperative outcomes, postoperative complications (such as bleeding events, recurrent laryngeal nerve injury, hypocalcemia), pathological findings, readmission, and follow-up outcomes. *Results*: Seventeen patients were included in this study, with female predominance (82.4%). The mean age was 85.6 ± 4.8 years. Fourteen patients (82.4%) exhibited compression-related symptoms as surgical indications. Based on pathological reports, patients were categorized into benign (12/17, 70.6%) and malignancy (5/17, 29.4%) groups. The benign group had a shorter operation time compared with the malignancy group (164.3 ± 32.0 min vs. 231.0 ± 79.1 min, *p* = 0.048). No major postoperative complications developed. The median postoperative follow-up duration was 28 months (range: 2–91 months). Thirteen patients (76.5%) were alive at the end of the study period. *Conclusions*: Despite potential age-related risks, thyroidectomy is feasible for carefully selected patients aged 80 years and older. It provides benefits not only in terms of oncological curative treatment but also in improving the quality of life, such as compressive symptoms and wound condition.

## 1. Introduction

As disease prevention and medical advancements continue, physicians will be required to provide care to the growing older patient population. In 2020, 9% of the global population was aged over 65 years old. According to the population projection report of Taiwan, it is estimated that by 2025, the country will have a super-aged society, with individuals aged over 65 accounting for more than 20% of the total population [1]. The global older population is expected to increase more than twofold in 2050 [2].

Thyroid nodules represent a prevalent condition in the general population, with a prevalence increasing with age [3]. Older adults also have a higher prevalence of high-risk thyroid cancer, as well as an increased risk of multimorbidity, functional decline, and postoperative complications [3,4].

As society continues to age, the proportion of older patients with thyroid tumors also increases. Understanding the risks and outcomes associated with thyroid surgery in these patients is crucial. 

Thyroid surgery in older patients is mainly performed in cases suspected of malignancy, thyrotoxicosis, or compressive symptoms. However, patients with difficulty in breathing or shortness of breath often first seek examination by a cardiologist or pulmonologist. This can inadvertently contribute to diagnostic misdirection, as the potential underlying thyroid disorders may remain unrecognized. 

Clinically, we often encounter patients or their families expressing concerns about the limited remaining lifespan, which leads to hesitancy regarding surgical interventions for those over 80 years. Therefore, to ensure that our findings are more applicable to the clinical setting, we chose a cutoff age of 80 years for our analysis. Additionally, the existing data are limited and have shown conflicting results, and data on Asian populations are lacking [5,6,7,8,9,10]. Therefore, we aimed to evaluate the detailed characteristics and outcomes of patients aged 80 years and older who underwent thyroid surgery.

## 2. Materials and Methods

### 2.1. Patient Population and Study Design

In this retrospective observational cohort study, patients who underwent thyroid-related surgery at our hospital between January 2015 and December 2022 were identified using the operation schedule system in the department’s database. Surgery for unilateral and bilateral total thyroidectomy was included. Patients were included if they were aged 80 years and older while undergoing thyroidectomy. A total of nineteen patients were included in the study. No patients with anaplastic carcinoma that underwent surgery were identified. After reviewing their medical records, two patients with thyroid cancer recurrence and those who underwent neck lymph node dissection without thyroidectomy were excluded. Subsequently, we selected patients with malignant pathological reports for further data analysis. This study ended in June 2023.

### 2.2. Thyroidectomy Protocol

All patients underwent preoperative neck ultrasonography. Patients identified by the anesthesiology department as having higher surgical risks (such as American Society of Anesthesiologists [ASA] classification over class 2 and history of heart disease) were scheduled for preoperative echocardiography to assess cardiac function. If the preoperative electrocardiogram and echocardiography showed any abnormalities, the patient was referred to a cardiovascular specialist for further evaluation. In cases where percutaneous coronary intervention or stent placement was needed, the operation was delayed according to guideline recommendations. All patients’ blood pressure was typically managed to meet the standards of maintaining systolic blood pressure (SBP) below 180 mmHg and diastolic blood pressure (DBP) below 110 mmHg before surgery.

Patients presenting with airway symptoms, such as wheezing or difficulty in breathing, were scheduled for a pulmonary function test to assess lung capacity and function.

All thyroidectomies were performed by each of the two experienced endocrine surgeons in our department. One attending surgeon has 13 years of experience, with a thyroidectomy volume of approximately 250 patients annually and treated 9 patients in this study. The other surgeon has 15 years of experience, with a thyroidectomy volume of approximately 180 and treated 8 patients in this study. During surgery, intraoperative neurophysiological monitoring (IONM) equipment and an energy device (HARMONIC^®^ scalpel, Ethicon, Cincinnati, OH, USA, or LigaSure™ sealer, Valleylab, Boulder, CO, USA) were used routinely, unless the patient could not afford the cost.

On the day after total thyroidectomy, both surgeons checked serum total calcium concentration to see if signs and symptoms of hypocalcemia were observed. One surgeon routinely monitored serum total calcium concentration on operation day and postoperative day 1, whereas the other surgeon did so only in response to symptomatic patients. Prophylactic calcium supplementation was not routinely administered following total thyroidectomy. It was only provided when blood test indicated hypocalcemia (serum total calcium concentration lower than 8.5 mg/dL) or when signs and symptoms of hypocalcemia, such as tingling or muscle cramps were reported. If no complications developed, the patients were discharged and followed up in the outpatient department two weeks after surgery. During follow-up, patients were evaluated for improvement of compression symptoms, surgical site healing, thyroid function, electrolyte conditions, and any delayed complications.

### 2.3. Clinical and Pathologic Characteristics

Clinical characteristics, including age, sex, body mass index (BMI), family history of thyroid disease, functional status, comorbidities, modified frailty index (mFI) score, history of other cancers, compression symptoms, preoperative image evaluation, ASA classification, size of the dominant tumor, fine-needle aspiration cytology (FNAC) results, use of IONM or energy devices, operation time, intraoperative blood loss, length of postoperative stay, in-hospital complications, postoperative survival time, and follow-up period, were collected. 

The patients’ preoperative functional status was classified into three categories, independent, partially dependent, and totally dependent, as assessed 30 days prior to the operation. Independence means that no assistance is required for daily activities, even with prosthetics or devices. Partial dependence refers to the need for assistance in daily activities, regardless of the use of equipment or aids [11]. Chronic kidney disease (CKD) was defined as an estimated glomerular filtration rate (eGFR) below 60 mL/min/1.73 m^2^ and categorized by stage according to the KDIGO guideline. Compression symptoms, including palpable mass, foreign body sensation, difficulty in breathing, dysphagia, and voice change, were analyzed. The size of the dominant tumor was determined using neck ultrasonography or computed tomography (CT). The results of TI-RADS (Thyroid Imaging Reporting and Data System) were collected. FNAC results were based on the Bethesda System for Reporting Thyroid Cytopathology. 

The duration of surgery was defined as the time from the initial skin incision to the completion of skin closure. The weight and volume of the excised thyroid glands were evaluated based on pathology reports. The events of postoperative intensive care unit (ICU) stay, delayed extubation, and blood transfusions were recorded. Postoperative complications, such as bleeding events, recurrent laryngeal nerve (RLN) injury, hypocalcemia, wound infection, pneumonia, cardiovascular events, and mortality, were recorded. Return to the emergency department and the need for readmission within 30 days of the procedure were recorded. The classifications of the thyroid pathologies were based on the final permanent pathological reports, which were reviewed by experienced pathologists. The benign thyroid pathology included nodular goiter, follicular adenoma, follicular neoplasm, and follicular nodular disease. The malignant tumors included papillary thyroid carcinoma, poorly differentiated carcinoma, Hürthle cell carcinoma, and follicular carcinoma. 

This study was approved by the Institutional Review Board of Taichung Veterans General Hospital (TCVGH-IRB No. CE23063C, 7 March 2023) and ended on 30 June 2023.

### 2.4. Statistical Analysis

Statistical analyses were performed using SPSS ver. 22 (IBM Corp., Armonk, NY, USA). Chi-square or Fisher’s exact tests were used to analyze categorical variables. For continuous variables, the Shapiro–Wilk test was used to test the normality of the sample distribution. A paired *t*-test or Mann–Whitney U test was used to compare continuous variables. Statistical significance was set at *p* < 0.05.

## 3. Results

### 3.1. Clinical Characteristics of the Patients before Thyroidectomy

In this retrospective cohort study, 17 patients aged 80 years and older were analyzed. 

Benign disease was found in 12 of 17 (70.6%) patients, and malignancy was confirmed in five (29.4%) patients. Based on the pathological report, the patients were divided into two groups: benign and malignant. The clinical characteristics of each group are summarized in Table 1. The mean age was 85.6±4.8 years, with a notable female predominance (14 patients, 82.4%). Patients with benign diseases exhibited a mean age of 84.4 ± 2.9 years, which was lower than that of patients with malignant diseases (88.4 ± 7.4 years). However, this difference was not significant (*p* = 0.339). 

The functional status of most patients was independent (10 patients, 58.8%), followed by partially dependent (7 patients, 41.2%). None of the patients were completely dependent.

The participants exhibited chronic comorbid conditions, including hypertension in 12 patients (70.6%) and CKD in 9 patients (52.9%, Supplementary Table S1). Chronic anemia, type 2 diabetes mellitus (DM), history of other cancers, and chronic pulmonary disease were observed in 35.3%, 30.4%, 17.6%, and 11.8% of patients, respectively. One patient had end-stage renal disease and underwent hemodialysis. 

Fourteen (82.4%) patients exhibited compression-related symptoms. Palpable masses or foreign body sensations (64.7%) and difficulty in breathing (52.9%) were the most frequently reported. Two patients (11.8%) experienced significant voice changes. 

The TI-RADS classification results and percentage of malignant pathology for the study population indicated that one out of eight patients (12.5%) in the TR2 group, three out of four patients (75%) in the TR3 group, and one out of five patients (20%) in the TR4 group had a malignant result, respectively.

More than half of the patients underwent neck CT (58.8%) and cardiac echocardiography (64.7%) for preoperative evaluation. Five (29.4 %) patients underwent lung function tests. Eleven patients were classified as ASA grade III (64.7%), and six patients were classified as ASA grade II (35.3%). The median mFI score of our patients was 0.2 (range, 0–0.4) and showed no difference between both groups.

Fifteen (88.2 %) patients underwent FNAC for preoperative diagnosis. Ten patients (66.7%) underwent unilateral aspiration, and the remaining underwent bilateral aspiration. FNAC was performed on 59 nodules across all patients. One (1.7%) major complication was severe hematoma with airway obstruction.

Based on the preoperative FNAC examinations using the Bethesda System for Reporting Thyroid Cytopathology, the incidence rates of non-diagnostic, benign, atypical, and malignant tumors were 26.7%, 53.3%, 13.3%, and 6.7%, respectively. The mean size of the dominant tumor was 5.5 ± 2.8 cm. The largest tumor was 10.6 cm. 

### 3.2. The Surgical Outcome of the Patients after Thyroidectomy

The surgical outcomes are summarized in Table 2. Unilateral total thyroidectomy was performed in 13 patients (76.4%); the remaining patients underwent bilateral total thyroidectomy (23.6%). IONM and energy devices were applied in 12 patients (70.6%). 

The mean operation time for the benign group was 164.3 ± 32.0 min, which was notably shorter than the 231.0 ± 79.1 min observed in the malignant group (*p* = 0.048). The mean estimated blood loss for the benign group was 33 ± 75 mL, which is lower than the 192 ± 189 mL observed in the malignancy group. However, this difference was not significant (*p* = 0.12). The median length of postoperative stay was 2 days (range, 1–15 days).

No postoperative bleeding events, RLN injuries, wound infections, or in-hospital mortality occurred in this cohort study. Two of the seventeen patients (11.8%) experienced transient hypocalcemia. None of the patients had surgery-related pulmonary complications, such as pneumonia or respiratory failure. Two patients required ICU stay, delayed extubation, and blood transfusion (11.8%). The reason for ICU admission was unrelated to thyroid surgery. One patient developed hematoma and respiratory failure after a FNAC test, which necessitated ICU admission before surgery. The other patient had a large ulcerative thyroid tumor with neck cellulitis and underwent total thyroidectomy with a left pectoralis major myocutaneous (PMMC) flap for wound reconstruction. For safety considerations, postoperative ICU admission, blood transfusion, and monitoring the condition of the flap were necessary.

One patient (5.9%) visited the emergency room within 30 days of the operation, which was attributed to the acute exacerbation of chronic obstructive pulmonary disease caused by pneumonia. The other patients were not readmitted postoperatively. One patient with dementia visited the emergency room 60 days after surgery due to poor medication compliance, which led to hypothyroidism.

Until the end of the study in June 2023, the median postoperative follow-up duration was 28 months (range: 2–91 months). Thirteen (76.5%) patients were alive at the end of the study period. Two patients died of pneumonia (not surgery-related) at 18 and 46 months postoperatively; one patient died from newly diagnosed adenocarcinoma of an unknown primary site with liver and lung metastases, which was diagnosed 1 month postoperatively and died due to rapidly progressive disease 2 months postoperatively; one patient died from frailty 9 months postoperatively. These four patients had a median follow-up duration of 13.5 months (range, 2–46 months). 

The clinicopathological results of five patients in the malignant group are summarized in Table 3 and Table 4.

### 3.3. Two Reports of Rare Presentation

Two patients aged 98 and 92 years had rare presentations before thyroidectomy.

#### 3.3.1. Case 1

The oldest patient was a 98-year-old woman with a 15-year history of hyperthyroidism, managed with carbimazole and bisoprolol. She had underlying hypertension, type 2 diabetes mellitus, and chronic kidney disease and was totally independent, with her daily living activities only assisted by a walker. The initial symptoms of neck swelling and palpitations appeared when she was in her 80s. Since then, she has received medical treatment for hyperthyroidism and multinodular goiter. Surgical intervention was suggested; however, her family refused due to her old age.

Six months prior to surgery, the patient experienced progressive worsening of symptoms, including dysphagia, odynophagia, and hoarseness. Thyroid ultrasonography revealed multiple bilateral nodules and mixed solid cystic calcifications. An endocrinologist then performed FNAC on 26 October 2021. Subsequently, the patient experienced neck swelling, pain, and stiffness after 5 min. Follow-up sonography revealed a hematoma at the puncture site without active bleeding. Compression was applied, and monitoring in the emergency department was recommended. However, the patient went home after 40 min of compression.

Later that day, she experienced difficulty in breathing and loss of consciousness at home. Cardiac massage was briefly performed by her son, after which her consciousness was regained. Upon arrival at the nearest emergency department, her Glasgow Coma Scale score was E4V1M1; endotracheal intubation was performed because of airway compression. Subsequently, the patient was transferred to the ICU of a local hospital.

**Figure 1:**
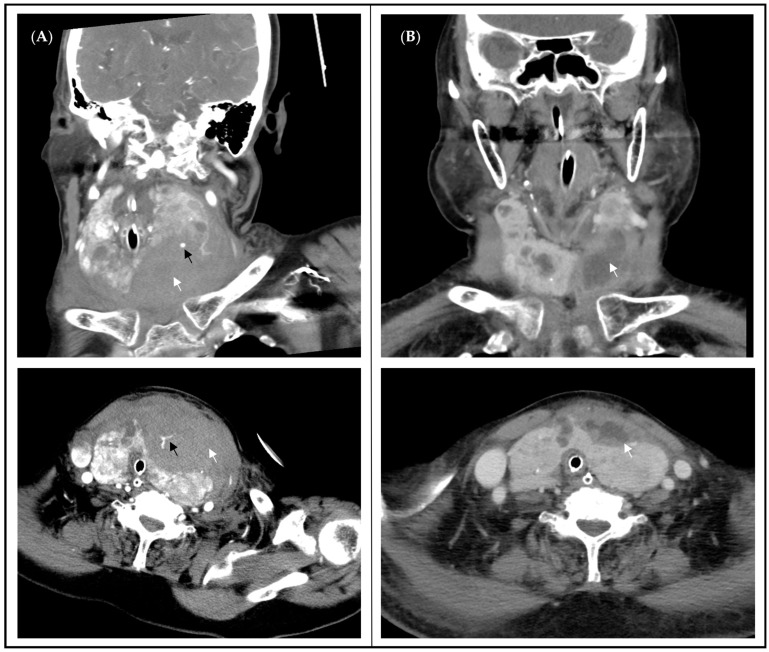
A 98-year-old woman developed hematoma after FNAC. (**A**): The neck CT showed enlargement of bilateral thyroid glands with multiple nodules, active bleeding (black arrow), and hematoma (white arrow), causing upper airway and left jugular vein compression with tracheal deviation. (**B**): The follow-up CT scan two weeks after FNAC showed hematoma (white arrow) in regression.

The neck CT scan on the day of admission revealed bilaterally enlarged thyroid glands (Figure 1A). The right-side thyroid measured 4.8 × 3.4 × 6.7 cm, and the left-side thyroid measured 3.9 × 4.6 × 9.1 cm, in addition to multiple nodules, intrathoracic extension, active bleeding, and a hematoma measuring 5 × 2.3 × 4.1 cm in the left neck. Surgical intervention was not recommended by the surgeon, and tracheostomy was suggested because of the difficulty in weaning from ventilator support. However, her family opted to transfer her back to our hospital on 11 November 2021 for surgical evaluation.

A subsequent neck CT scan performed at our hospital revealed a regressing hematoma (Figure 1B). Thirty-four days after fine-needle aspiration, left thyroidectomy was performed for airway decompression on 19 November 2021, and the patient remained in the ICU for postoperative care. The endotracheal tube was removed on postoperative day 5. Fifteen days postoperatively, the patient was discharged in a stable condition. Pathological examination revealed nodular goiter and papillary microcarcinoma. Follow-up in the outpatient department confirmed that the patient had maintained euthyroid status.

#### 3.3.2. Case 2

A 92-year-old woman with a decades-long history of goiter was initially diagnosed at another hospital; the exact FNAC report remains uncertain owing to a lack of available records. Surgical intervention was proposed as a management strategy, but the patient declined it. Since then, the patient has been lost to follow-up.

**Figure 2:**
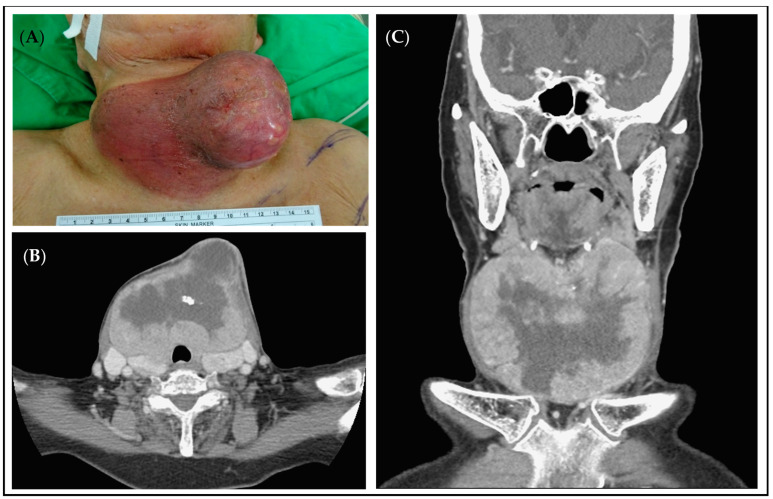
(**A**): A 92-year-old woman suffered from a large thyroid tumor with skin ulceration and cellulitis. (**B**,**C**): The neck CT scan showed an enhancing necrotic mass 10 × 11 × 10 cm with calcification in lower neck submental space, abutting bilateral thyroid glands.

Before the operation, she experienced progressive enlargement of the neck mass and a painful sensation associated with skin ulceration for 1 month. The neck CT scan performed on 2 February 2021 revealed a thyroid lesion measuring 8.8 × 9.1 × 10.6 cm with skin ulceration (Figure 2). The patient was admitted for the management of cellulitis and the neck mass.

**Figure 3:**
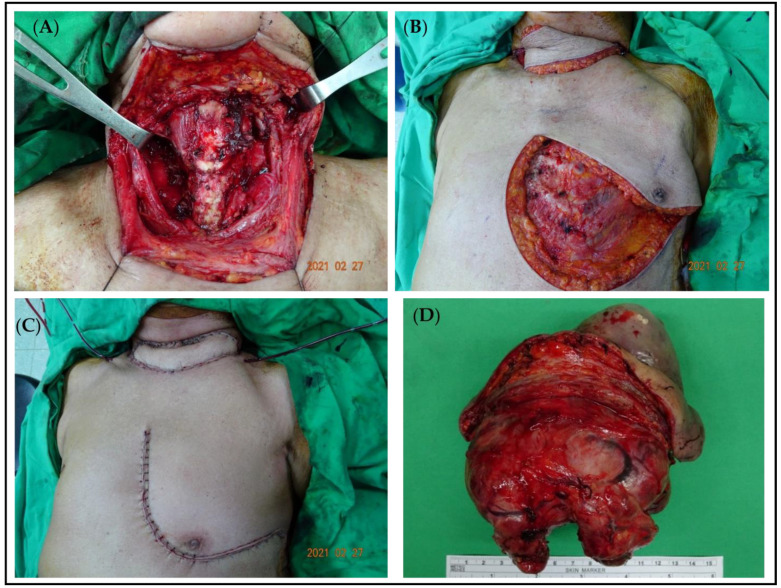
(**A**): The wound of a 92-year-old woman with a large thyroid tumor after total thyroidectomy. (**B**,**C**): The wound before and after PMMC flap for wound reconstruction. (**D**): A soft tumor, 8 × 5 × 3.2 cm, with invasion into bilateral lobes of thyroid and skin tissue.

The FNAC test of the neck mass revealed thyroid squamous cell carcinoma (SCC). Considering the significant size of the tumor and skin involvement, total thyroidectomy with a left PMMC flap for wound reconstruction was performed on 27 February 2021. (Figure 3).

Postoperatively, the patient was transferred to the ICU care unit to monitor the flap condition. Extubation was delayed until postoperative day 5 because of the neck wound swelling, which may cause respiratory failure. Thirteen days postoperatively, the patient was discharged in a stable condition. The final pathological result was poor-to-undifferentiated carcinoma with prominent squamous differentiation.

## 4. Discussion

In this retrospective study, the overall patient cohort comprised 82.4% women, with a malignancy rate of 29.4%, consistent with the findings of several large-scale analyses [7,8,12]. The mean age of the patients was 85.6 years, which is higher than the average life expectancy in Taiwan (80.86 years) [13].

As the population continues to age, the frequency of surgical procedures involving older individuals is expected to increase. The diagnosis and treatment protocols for thyroid nodules are not influenced by age; therefore, the number of older patients requiring these surgical procedures is likely to increase. 

Compression-related symptoms were the most common indications for thyroidectomy in this study. Fourteen (82.4%) patients underwent thyroidectomy to treat compression-related symptoms. Additionally, one patient underwent thyroidectomy due to post-FNAC complications, including hematoma and respiratory failure. Another study demonstrated that common surgical indications in older patients include hyperthyroidism resistant to medical management, symptoms of compression, and suspicion of malignant nodules [14]. Four of the patients underwent bilateral thyroidectomy, and the other ten underwent unilateral thyroidectomy. David et al. demonstrated that unilateral and bilateral thyroidectomy are equally effective for the relief of compressive symptoms [15]. Shared decision-making allows patients to choose the surgical procedure after being informed of the risks and benefits associated with both.

The study shows a 26.7% rate of non-diagnostic FNAC results. Given the retrospective nature of this study, data limitations make it difficult to determine the exact reasons. However, possible causes could include insufficient sampling or challenges in sampling larger or more complex cystic tumors.

The median time from the diagnosis of a thyroid tumor to thyroidectomy was 12 months (range, 1 month to 40 years). Concerns regarding potential postoperative complications among older patients and their families have led to considerable delays in the decision to undergo surgery. This could be attributed to the high incidence of complications observed in patients aged over 65 years, as reported in previous studies [7,8].

The incidence of perioperative cardiac complications is high in older patients aged over 80 years with coronary artery disease [16]. Preoperative electrocardiogram and echocardiography may provide important information for patients at an increased risk of cardiac complications [17]. To date, our hospital has updated preoperative protocol, and all patients aged 80 years and older routinely undergo preoperative echocardiography. 

The average duration of our thyroidectomy procedures was 183.9 ± 57.0 min. The malignancy group (231.0 ± 79.1 min) experienced significantly longer operation times compared to the benign group (164.3 ± 32.0 min) (*p* = 0.048). However, owing to the limited sample size, these differences did not achieve significance in the unilateral and bilateral thyroidectomy subgroup analyses (*p* = 0.143, *p* = 1). The increased operation time in the malignant group may be due to extensive tissue invasion requiring resection and wound reconstruction, premalignant desmoid reaction, tumor adherence to adjacent structures with complicated dissection, and enhanced vascularity demanding meticulous hemostasis. Another explanation for this difference is that more patients with benign conditions underwent unilateral thyroidectomy (83.3%) than those with malignant conditions (60%), although this difference was not significant (*p* = 0.163).

In our study, the size of the dominant thyroid tumor was 5.5 ± 2.8 cm. Dellal et al. compared thyroidectomy in patients aged ≥65 and <65 years old, revealing that the mean nodule diameter was significantly larger in geriatric patients [18]. Based on our clinical experience, the operative time of older patients was longer than those of younger patients, possibly due to larger tumor size. Further study is needed to examine the differences and associations between tumor size and operative times in older and younger patients (Supplementary Table S2).

A majority of post-thyroidectomy complications in our patient groups were transient hypocalcemia, which occurred in two patients (11.8%). Some older patients are less sensitive to the symptoms of hypocalcemia and are less likely to report symptoms. Routine monitoring of serum calcium levels within 12–24 h post thyroidectomy may alleviate hypocalcemia symptoms and enhance the overall safety of patients [19]. We have adapted to this protocol. In our study, no major complications such as bleeding, RLN injury, wound infections, pneumonia, cardiovascular events, in-hospital mortality, or instances of readmission and reoperation were reported. Postoperative complication rates in previous studies varied from 1.2% to 6.8% [7,8,9,10,12,20]. Moreover, recent research has suggested that the incidence of complications may increase among older patients [7,8]. Post-thyroidectomy hypocalcemia ranges from 6% to 16.2% [10,13]. In a large national dataset analysis, Sahli et al. showed that the overall complication rate after thyroidectomies was 1.3%, the need for reoperation was 0.8%, and the readmission rate was 2.3% [7]. Older age was associated with an increased overall risk of complications (OR = 2.67). This reduced major complication rate could be ascribed to the expertise of high-volume surgeons. Supporting this finding, other studies have indicated a notable decrease in both thyroid cancer recurrence rates and postoperative complications among patients treated by high-volume surgeons, underscoring the importance of surgical experience in improving patient outcomes [21,22].

Matched cohort studies conducted by Papoian et al. showed that a higher Charlson Comorbidity Index is associated with an elevated risk of postoperative complications [8]. However, calculating the Charlson Comorbidity Index is relatively complex. Frailty is associated with a higher incidence of complications and mortality [23,24]. Moreover, mFI is strongly correlated with increased rates of postoperative complications, readmission, reoperation, discharge to skilled care, longer hospital stays, and higher mortality rates. A meta-analysis conducted by Panayi et al. revealed that postoperative mortality was more prevalent among patients with frailty and an mFI score of 0.36 or higher compared to those with lower scores [24]. The mFI score is simpler to calculate using patient characteristics. In our retrospective statistical analysis, the median mFI score of the patients was 0.2 (range of 0–0.4). Careful selection of older patients for surgery can decrease postoperative complications.

One patient developed a hematoma after the FNAC test, which necessitated intubation for airway maintenance and ICU admission before surgery. The FNAC test is currently the simplest, safest, and most cost-effective method for the identification of malignant thyroid nodules. However, this may cause a hematoma and require further surgical treatment. In this study, a FNAC test was performed for 17 patients with 59 thyroid nodules. Major complications occurred in one patient (1.7%), which presented as a massive hematoma and caused airway obstruction. Cappelli et al. reported that the complication rate of fine needle aspiration was approximately 0.15%, and major complications were 0.075% [25]. In older patients with loose soft tissue, the use of antiplatelet medication increases the feeding vessels of tumors and may increase the risk of hematoma. Using color Doppler ultrasonography to detect hypervascularity in a thyroid tumor and scheduling the withholding of antiplatelet or anticoagulant medications may reduce the risk of post-procedural bleeding complications.

One patient with dementia visited the emergency department 2 months after undergoing total thyroidectomy due to the discontinuation of medication-related hypothyroidism. The age of older patients was associated with misunderstanding the medication and medical treatment, eventually decreasing compliance [26]. The prevalence of dementia also increases with age and worsens comprehension and compliance with medical treatment. Individual decision-making on prescription appropriateness in patients with dementia and closely monitoring their understanding of medication could enhance medication adherence and potentially reduce emergency department visits [27]. The case manager's diligent follow-up with the patient and reminders to caregivers could enhance medication compliance. Unilateral thyroidectomy in selected patients with thyroid cancer may prevent hypothyroidism and improve the quality of life of older patients [28]. Shared decision-making also plays a crucial role in the choice between unilateral or total thyroidectomy, not only offering benefits in the quality of life and oncological outcomes but also alleviating the burden on patients and caregivers.

Although our study provides an in-depth analysis of outcomes in patients aged 80 years and older, it has certain limitations. The results were based on the experience of a single center with only 17 patients. While we recorded the presence of chronic diseases, we did not collect detailed data on preoperative blood pressure levels or control status, which may influence perioperative and postoperative outcomes. Future studies that incorporate more comprehensive data collection in these areas would help clarify their impact on patient outcomes. The feedback on quality of life we received from patients and their families was valuable; it was inherently subjective and based on observational data rather than quantitative measures. Future studies using multicenter or national data banks would provide stronger and more complete evidence.

## 5. Conclusions

Thyroidectomy is feasible for carefully selected patients aged 80 years and older. It provides benefits not only in terms of oncological curative treatment but also in improving the quality of life, such as compression symptoms and wound condition.

## Figures and Tables

**Table 1:** Preoperative clinical characteristics of patients aged 80 years and older.

Characteristics	Overall (N = 17)	Benign (N = 12)	Malignant (N = 5)	*p*-Value
Age ^†^ (years)	85.6 ± 4.8	84.4 ± 2.9	88.4 ± 7.4	0.339
Sex, female, n (%)	14 (82.4%)	10 (83.3%)	4 (80%)	1.000
BMI ^†^	24.5 ± 4.3	24.2 ± 4.9	25.1 ± 2.4	0.383
Family history of thyroid disease, n (%)	1 (6%)	0	1 (20%)	0.294
History of thyroidectomy	2 (11.8%)	1 (8.3%)	1 (20%)	0.515
Functional status, n (%)				1.000
Independent	10 (58.8%)	7 (58.3%)	3 (60%)	
Partially dependent	7 (41.2%)	5 (41.7%)	2 (40%)	
Totally dependent	0	0	0	
Comorbidity, n (%)				
Type 2 Diabetes mellitus	5 (30.4%)	2 (16.7%)	3 (60%)	0.117
Hypertension	12 (70.6%)	8 (66.7%)	4 (80%)	1.000
Cardiac vascular disease	2 (11.8%)	1 (8.3%)	1 (20%)	0.515
Chronic kidney disease	9 (52.9%)	7 (58.3%)	2 (40%)	0.620
Chronic pulmonary disease	2 (11.8%)	2 (16.7%)	0	1.000
Chronic anemia	6 (35.3%)	3 (25%)	3 (60%)	0.280
Other cancers	3 (17.6%)	2 (16.7%)	1 (20%)	1.000
Modified Frailty Index ^†,††^	0.16 ± 0.12 (0.2 [0–0.4])	0.15 ± 0.12 (0.15 [0–0.4])	0.18 ± 0.11 (0.2 [0–0.3])	0.574
All compression symptoms, n (%)	14 (82.4%)	10(83.3%)	4 (80%)	1
Palpable mass or foreign body sensation	11 (64.7%)	7 (58.3)	4 (80%)	0.600
Difficulty in breathing	9 (52.9%)	6 (50%)	3 (60%)	1.000
Dysphagia	6(35.3%)	4 (33.3%)	2 (40%)	1.000
Voice change	2 (11.8%)	1(9.3%)	1 (20%)	0.515
Preoperative evaluation, n (%)				
Neck CT scan	10 (58.8%)	6 (50%)	4 (80%)	0.338
TI-RADS T1/T2/T3/T4/T5, n	0/8/4/5/0	0/7/1/4/0	0/1/3/1/0	0.667
Echocardiography	11 (64.7%)	7 (58.3)	4 (80%)	0.600
Lung function test	5 (29.4%)	3 (25%)	2 (40%)	0.472
ASA-1/2/3/4, n	0/6/11/0	0/4/8/0	0/2/3/0	0.605
Dominant tumor size ^†^ (cm)	5.5 ± 2.8	5.5 ± 2.3	5.6 ± 4.2	1
FNAC	15 (88.2%)	11 (91.7%)	4 (80%)	0.447
Time ^††^	1 [0–17]	1 [0–17]	2 [0–9]	1.000
Unilateral %	10 (66.7%)	8 (72.7%)	3 (60%)	0.407
Bilateral %	5 (33.3%)	3 (27.2%)	2 (40%)	0.407
Bethesda category				
Nondiagnostic	4 (26.7%)	2 (16.7%)	2 (40%)	
Benign	8 (53.3%)	8 (66.7%)	0	
Atypia	2 (13.3%)	1 (8.3%)	1 (20%)	
Suspicious for malignancy	0	0	0	
Malignancy	1 (6.7%)	0	1 (20%)	

Data presented as ^†^ Mean ± SD, ^††^ Median (range). CT—computed tomography; FNAC—fine-needle aspiration cytology.

**Table 2:** Surgical outcomes of patients aged 80 years and older.

Characteristics	Overall (N = 17)	Benign (N = 12)	Malignant (N = 5)	*p*-Value
Surgery				0.538
Unilateral thyroidectomy, n (%)	13 (76.4%)	10 (83.3%)	3 (60%)	
Bilateral thyroidectomy, n (%)	4 (23.6%)	2 (16.7%)	2 (40%)	
IONM, n (%)	12 (70.6%)	8 (66.7%)	4 (80%)	1.000
Energy devices, n (%)	12 (70.6%)	8 (66.7%)	4 (80%)	1.000
Operation time †,†† (min)	183.9 ± 57.0 (180 [130–350])	164.3 ± 32.0 (157.5 [130–230])	231.0 ± 79.1(200 [155–350])	0.048
Unilateral thyroidectomy (min)	172.0 ± 43.3 (155 [130–270])	161.1 ± 34.4 (145 [130–230])	208.3 ± 58.0(200 [155–270])	0.143
Bilateral thyroidectomy (min)	222.5 ± 85 (222 [180–350])	180.0 ± 0(180 [180–180])	265.0 ± 120.2(265 [180–350])	1.000
Blood loss †,†† (mL)	80 ± 135 (0 [0–400])	33 ± 75 (0 [0–250])	192 ± 189 (210 [0–400])	0.12
Thyroid volume *,†,†† (mL)	97.9 ± 86.1 (86.2 [5.5–284.5]	91.9 ± 82.6 (71.9 [14.7–284.5])	112.5 ± 102.7 (103.5 [5.5–227.8])	0.861
Each Thyroid volume ***,†,†† (mL)	97.9 ± 86.1 (60.4 [5.5–284.5]	78.7 ± 75.2 (58.9 [14.7–284.5])	69.1 ± 52 (103 [5.5–113.9])	0.820
Unilateral thyroidectomy †,†† (mL)	70.3 ± 76.4 (57.5 [5.5–284.5])	78.6 ± 82.6 (57.5 [14.7–284.5])	42.9 ± 53 (19.6 [5.5–103.5])	0.469
Bilateral thyroidectomy †,†† (mL)	187.7 ± 46.6 (201.2 [120.7–227.8])	158.5 ± 53.5 (158.5 [120.7–196.4])	216.9 ± 15.4 (216.9 [206–227.8])	0.333
Thyroid weight †,††,** (g)	117.3 ± 131.2 (90 [10–510])	96.0 ± 84.6 (90 [10–300])	160.0 ± 201.7 (110 [10–510])	0.743
Each thyroid weight **** (g)	88.3 ± 85.6 (65 [10–300])	84.5 ± 85.2 ([10–300])	69.1 ± 52 (65 [10–255]	0.979
Unilateral thyroidectomy †,†† (g)	80.9 ± 84.8 (70 [10–300])	91.3 ± 95.2 (80 [10–300])	53.3 ± 51.3 (40 [10–110])	0.673
Bilateral thyroidectomy †,†† (g)	217.5 ± 195.2 (125 [110–510])	115.0 ± 7.1 (115 [110–120])	320.0 ± 268.7 (320 [130–510])	0.333
Length of stay †,†† (days)	3.1 ± 4.2 (2 [1–15])	1.7 ± 0.8 (1.5 [1–3])	6.6 ± 6.8 (2 [1–15])	0.134
Time to operation †,†† (months)	98.9 ± 143.3 (12 [1–500])	114.6 ± 162.5 (42 [2–500])	61.4 ± 83.6 (3 [1–180])	0.737
Postoperative follow-up time †,†† (months)	33.2 ± 27.6 (28 [2–91])	32 ± 28 (24.5 [2–91])	36 ± 29.5 (28 [9–85])	0.646

Data presented as ^†^ Mean ± SD, ^††^ Median (range). * Total thyroid volume was calculated as length × width × height × 0.479. ** Two patient’s thyroid weight data were lost. *** Each thyroid volume was calculated as length × width × height × 0.479/Ɵ; Unilateral thyroidectomy Ɵ = 1, Bilateral thyroidectomy Ɵ = 2. **** each thyroid weight was calculated as thyroid weight/Ɵ. IONM—intraoperative neurophysiological monitoring.

**Table 3:** Clinical information of the five patients with malignancy.

Patient No.	Age	Sex	Functional Status	Comorbidity	mFI	Indication for Surgery	Patient Referred from Specialist	FNAC	Operation	Operation Time (min)	Postoperative Complication	Postoperative Stay (Days)	Follow-Up Time (Months)
1	98	F	independent	DM, HTN, CKD, Hyperthyroidism	0.2	Hematoma after FNAC caused respiratory failure	Endocrinologists	Non-diagnostic	Left thyroidectomy	270	nil	15 *	19 ^ǝ^
2	92	F	dependent	HTN	0.2	Rapid progression of neck mass with skin ulceration	Endocrinologists	Squamous cell carcinoma	Total thyroidectomy + PMMC flap	350	Transient hypocalcemia	13 *	28 ^ǝ^
3	90	M	independent	DM, HTN, CKD, Urothelial carcinoma	0.2	Difficulty in breathing and mass sensation	Urologist	Benign	Total thyroidectomy	180	nil	2	9 ^※^
4	82	F	independent	nil	0	Multiple pulmonary nodules post wedge resection with pathologic confirmation of metastatic thyroid carcinoma.	Thoracic surgeon	nil	Right thyroidectomy ^Ɵ^	155	Transient hypocalcemia	1	85 ^ǝ^
5	80	F	dependent	DM, HTN, Hyperthyroidism, Parkinsonism, atrial fibrillation	0.3	Chest tightness, difficulty in breathing	Endocrinologists	atypia cell	Left thyroidectomy ^Ɵ^	200	nil	2	39 ^ǝ^

* ICU admission; ^※^ death due to frailty; ^ǝ^ remained alive until the end of the study; ^Ɵ^ history of thyroidectomy. CKD—chronic kidney disease; HTN—hypertension; DM—diabetes; FNAC—fine-needle aspiration cytology; PMMC—pectoralis major myocutaneous.

**Table 4:** Pathological results of the five patients with malignancy.

Patient No.	Age	Sex	Pathology	Associated Benign Thyroid Disease in Pathology	Dominant Tumor in Image Size (cm)	Pathological Cancer Size (cm)	Thyroid Weight (g)	Thyroid Volume(mL)	Multifocal	Angioinvasion	Lymphatic Invasion	Perineural Invasion	Extrathyroidal Extension
1	98	F	papillary microcarcinoma	Nodular goiter	4.6	0.2	110	103.5	Unifocal	nil	nil	nil	nil
2	92	F	poorly to undifferentiated carcinoma with prominent squamous differentiation, bilateral	Follicular neoplasm of uncertain malignant potential, left	10.6	8	510	227.8	Unifocal	nil	nil	nil	Present
3	90	M	Hürthle cell carcinoma, left	Nodular goiter, right	9.3	9	130	206.0	Unifocal	nil	nil	nil	nil
4	82	F	Papillary carcinoma, follicular variant, right	nil	0.5	0.5	10	5.5	Unifocal	nil	nil	nil	nil
5	80	F	Follicular carcinoma, encapsulated angioinvasive, with focal poorly differentiated thyroid carcinoma (5%)	nil	3.9	3.2	40	19.6	Unifocal	Present	nil	nil	nil

## Data Availability

The data presented in this study are available upon reasonable request from the corresponding authors (W.H. or W.-H.C).

## Supplementary Materials

The following supporting information can be downloaded at: https://www.mdpi.com/article/10.3390/medicina60091383/s1, Table S1: Renal Function Assessment in patients aged 80 years and older; Table S2: Correlation Analysis Between Operation Time and Various Factors.
