# Supplementary material for: Surgical Outcomes of Thyroidectomy in Geriatric Patients Aged 80 Years and Older: A Single-Center Retrospective Cohort Study
Source: Medicina (Kaunas). 2024 Aug 23;60(9):1383. doi: 10.3390/medicina60091383 (PMC11433778; doi:10.3390/medicina60091383)
Supplement: Supplementary file 1 [file medicina-60-01383-s001.zip › medicina-3140259-supplementary.pdf]

Supplementary Tables

Table S1. Renal Function Assessment in patients aged 80 years and older.

**Patient No.**	**Sex**	**Pathological Report**	**Age**	**eGFR ($ml/min/1.73m^2$)**
1	Female	Malignancy	98	77.12
2	Female	Malignancy	92	101.3
3	Female	Benign	90	31.73
4	Male	Malignancy	90	28.25
5	Female	Benign	87	45.27
6	Female	Benign	86	50.71
7	Female	Benign	86	55.24
8	Male	Benign	86	54.19
9	Female	Benign	85	75.72
10	Male	Benign	85	50.77
11	Female	Benign	84	82.02
12	Female	Malignancy	82	60.59
13	Female	Benign	82	105.8
14	Female	Benign	81	52.88
15	Female	Benign	81	85.36
16	Female	Benign	80	77.83
17	Female	Malignancy	80	55.42

eGFR calculated with MRDR formular = 175 x SerumCr-1.154 x age-0.203 x 1.212 (if patient is black) x 0.742 (if female) ml/min/1.73m2

Table S2. Correlation Analysis Between Operation Time and Various Factors.

**Operation Time vs. Variables**	**Correlation Coefficient (r-value)**	***p*-Value**
Age	0.389	0.123
BMI	0.373	0.14
eGFR	0.11	0.966
mFI	0.329	0.197
Tumor Size	0.329	0.197
Surgeon	N/A	0.963
Gender	N/A	1

Pearson correlation was used for normally distributed data, and Spearman correlation was applied where data did not meet normality assumptions.
